# Auxin-Inducible Depletion of the Essentialome Suggests Inhibition of TORC1 by Auxins and Inhibition of Vrg4 by SDZ 90-215, a Natural Antifungal Cyclopeptide

**DOI:** 10.1534/g3.118.200748

**Published:** 2019-01-22

**Authors:** Nathan A. Snyder, Adam Kim, Louis Kester, Andrew N. Gale, Christian Studer, Dominic Hoepfner, Silvio Roggo, Stephen B. Helliwell, Kyle W. Cunningham

**Affiliations:** * Department of Biology, Johns Hopkins University, 3400 N. Charles Street, Baltimore, Maryland 21218; † Novartis Institutes for BioMedical Research, Novartis Campus, CH-4056 Basel, Switzerland

**Keywords:** auxin, auxin inducible degron, functional genomics, glycosylation, Golgi, rapamycin

## Abstract

Gene knockout and knockdown strategies have been immensely successful probes of gene function, but small molecule inhibitors (SMIs) of gene products allow much greater time resolution and are particularly useful when the targets are essential for cell replication or survival. SMIs also serve as lead compounds for drug discovery. However, discovery of selective SMIs is costly and inefficient. The action of SMIs can be modeled simply by tagging gene products with an auxin-inducible degron (AID) that triggers rapid ubiquitylation and proteasomal degradation of the tagged protein upon exposure of live cells to auxin. To determine if this approach is broadly effective, we AID-tagged over 750 essential proteins in *Saccharomyces cerevisiae* and observed growth inhibition by low concentrations of auxin in over 66% of cases. Polytopic transmembrane proteins in the plasma membrane, Golgi complex, and endoplasmic reticulum were efficiently depleted if the AID-tag was exposed to cytoplasmic OsTIR1 ubiquitin ligase. The auxin analog 1-napthylacetic acid (NAA) was as potent as auxin on AID-tags, but surprisingly NAA was more potent than auxin at inhibiting target of rapamycin complex 1 (TORC1) function. Auxin also synergized with known SMIs when acting on the same essential protein, indicating that AID-tagged strains can be useful for SMI screening. Auxin synergy, resistance mutations, and cellular assays together suggest the essential GMP/GDP-mannose exchanger in the Golgi complex (Vrg4) as the target of a natural cyclic peptide of unknown function (SDZ 90-215). These findings indicate that AID-tagging can efficiently model the action of SMIs before they are discovered and can facilitate SMI discovery.

One of the most powerful approaches for experimental determination of gene function involves the identification of phenotypes that appear in the cells of interest upon introduction of mutations that decrease function. Such loss-of-function mutations can be produced easily in many eukaryotic cell types using CRISPR/Cas9-based technologies, and earlier methods involving homologous recombination, insertional mutagenesis, and random mutagenesis have been very useful in a wide variety of model organisms. Nearly complete arrays of gene knockout mutants have been produced in budding yeast *Saccharomyces cerevisiae* (Winzeler *et al.* 1999) and the fission yeast *Schizosaccharomyces pombe* (Kim *et al.* 2010), with several additional species of pathogenic fungi currently in progress (Roemer *et al.* 2003; Schwarzmüller *et al.* 2014; Liu *et al.* 2008). Though such collections offer enormous potential for understanding diverse biological processes, the general approach is hampered by the inability to knockout essential genes, which typically constitute 10–20% of the genome. Most essential genes in *S. cerevisiae* were successfully rendered hypomorphic by introducing knockout mutations in heterozygous diploids or by introducing mutations in the 3′ untranslated regions of haploids (Breslow *et al.* 2008). However, with these approaches the cells are studied long after the mutation was created, which makes discriminating primary defects from secondary adaptations very challenging. In addition to such epigenetic effects, secondary mutations often arise that compensate for or obscure the phenotypes of primary mutations (Teng *et al.* 2013).

Conditional knockout or knockdown of gene function can eliminate some of the major limitations of the unconditional gene knockouts described above. In *S. cerevisiae*, about 75% of essential genes have been mutated in such a way to render the protein product non-functional at high temperature but functional at low temperature (Ben-Aroya *et al.* 2008; Li *et al.* 2011). Such temperature-sensitive mutations allow easy and often reversible inactivation of gene function. However, they are relatively difficult to produce and often difficult to interpret because the level of gene function may be abnormal even at the permissive temperature and incompletely or slowly inactivated at the non-permissive temperature. Additionally, the temperature shifts themselves may cause undesirable biological consequences that could confound interpretations. Alternatively, essential genes can be placed under control of regulatory systems that enable tight shut-off of gene transcription (for example, glucose-, methionine-, and tetracycline-repressible promoters). Phenotypic analyses can then be made as the mRNA and protein products decay at their natural rates (Roemer *et al.* 2003). CRISPRi using dCas9 can achieve similar repression without altering gene sequences (Qi *et al.* 2013; Smith *et al.* 2017). Other approaches enable ligand-responsive de-capping, de-tailing, or translational frameshifting of targeted mRNAs (Klauser *et al.* 2015; Anzalone *et al.* 2016). These mRNA knockdown approaches may be combined for improved performance, but still the long cellular lifespans of many proteins will delay the appearance of phenotypes.

Several approaches have enabled rapid conditional destruction or mislocalization of targeted proteins. One approach involves N-terminal tagging of the proteins of interest with a temperature-sensitive degron that enables misfolding, ubiquitylation, and degradation of the fusion protein by the 26S proteasome (Dohmen and Varshavsky 2005). The tag itself allows quantitation of the rate and extent of protein destruction, but also may interfere to some extent with protein function even under the permissive condition.

Similarly, C-terminal tagging of proteins with the auxin-inducible degron (AID) sequence from plants can enable rapid ubiquitylation and proteasomal degradation of the protein upon addition of a small molecule auxin (indole-3-acetic acid) (Nishimura *et al.* 2009; Morawska and Ulrich 2013). This approach requires co-expression of an E3 ubiquitin ligase from plants such as OsTir1 that recognizes AID-tagged proteins bound to auxin. The AID-tagging and target depletion system works very well in plant, animal, and fungal cell types and shows great promise for functional genomics research (Natsume and Kanemaki 2017). However, this conditional degron technology has not yet been implemented genome-wide and its effectiveness and limitations are not fully known.

In this study, we AID-tag the C-termini of 758 essential and 313 non-essential gene products of *S. cerevisiae* and simultaneously introduce the OsTIR1 expression cassette together with a selectable marker. The effects of auxin and a non-metabolizable auxin analog (1-naphthaleneacetic acid; NAA) on cell growth were analyzed carefully. Surprisingly, we find that auxin and especially NAA have off-target inhibitory effects on the TORC1 protein kinase. At much lower concentrations, auxin depletes most AID-tagged proteins below the phenotypic threshold even if they spanned membranes of the endoplasmic reticulum, Golgi complex, plasma membrane, and mitochondrial outer membrane. We then explore the possibility that auxins synergize with other small molecules that are known to bind and inhibit particular target proteins. Finally, we show that an antifungal cyclopeptide SDZ 90-215 synergizes with auxin in *VRG4-AID* strains but not control strains and that amino acid substitutions within the substrate-binding pocked of Vrg4 protein confer strong resistance to the compound. These findings validate the use of AID-tagged strains for functional genomics research and for discovery of novel SMIs and antifungals.

## Materials and Methods

### Plasmids, Yeast strains, and genetic screens

**Figure 1:**
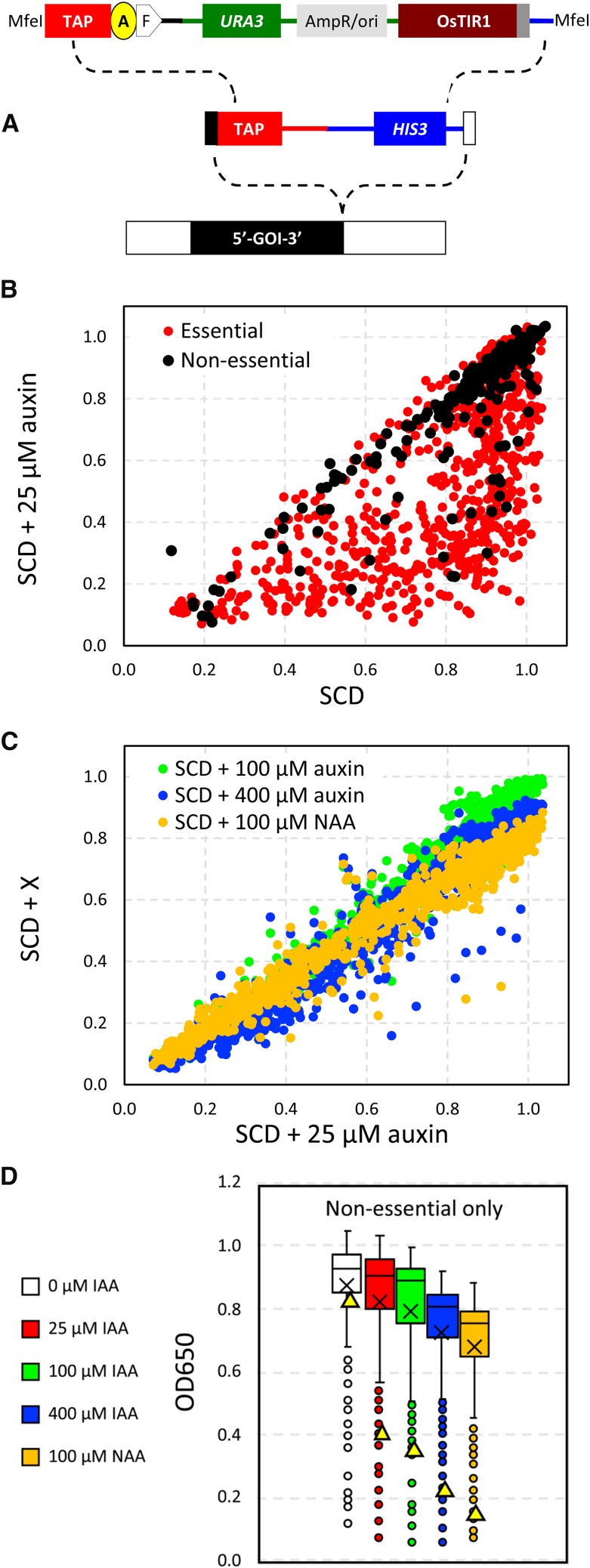
AID-tagging and growth defects caused by auxin and NAA. (A) Schematic of the MfeI-linearized pAIDA2(6FLAG) plasmid that was transformed into individual strains of *S. cerevisiae* with a TAP-tag already present in the gene of interest (GOI). The minimal AID-tag and 6xFLAG-tag are labeled “A” and “F” and the OsTIR1 expression cassette is also indicated. (B) Optical densities of 756 essential (red) and 315 non-essential (black) AID-tagged strains after diluting stationary phase 36-fold into fresh SCD medium with and without 25 µM auxin and incubating for 20 hr at 23°C. (C) Optical densities of AID-tagged strains after culturing in 100 or 400 µM auxin or 100 µM NAA as compared to 25 µM auxin. (D) Box plots of non-essential AID-tagged strains grown in different conditions (centerline = median, X = average, box = middle quartiles, whiskers = middle deciles, circles = outliers, triangles = *GTR1-AID* strain).

TAP-tagged strains of *S. cerevisiae* in the BY4741 background (Ghaemmaghami *et al.* 2003) were purchased from Dharmacon Inc., grown to log phase in YPD medium, transformed with the MfeI-digested linearized plasmid pAIDA2(6FLAG) depicted in Figure 1 using the lithium acetate method, and plated onto synthetic complete medium lacking uracil and containing 2% dextrose (SCD-ura agar medium) to select for transformants using conventional procedures (Sherman *et al.* 1986). From each transformation, four independent colonies were picked, purified by streaking onto SCD-ura agar medium, and tested for correct AID-tagging of by assessing growth on SCD-his agar medium. Three validated colonies from each transformation were then arrayed in 96-well culture dishes and tested for growth defects in SCD medium containing 100 µM auxin. The concordance rate among the three isolates was over 99%, and therefore a single representative was chosen and arrayed into the master set of 96-well dishes. The master set containing 15% glycerol was frozen and stored at -80°. Additional strains of *S. cerevisiae*, *C. glabrata*, and *C. albicans* are listed in Table 1.

**Table 1:** Yeast strains used in this study

NAME	GENOTYPE	REF
K1251	*MATa*	[23]
AK060	*MATa gtr1*::*NatR*	[23]
AK061	*MATa gtr2*::*NatR*	[23]
AK062	*MATa ego1*::*NatR*	[23]
AK063	*MATa ego3*::*NatR*	[23]
AK018	*MATa pib2*::*NatR*	[23]
AK104	*MATx TOR1(I954S)*	[23]
AK102	*MATx gtr1*::*NatR TOR1(I954S)*	[23]
AK106	*MATx pib2*::*KanR TOR1(I954S)*	[23]
AK108	*MATx gtr1*::*NatR pib2*::*KanR TOR1(I954S)*	[23]
NS270	*MATa LCB1-TAP-AID-6FLAG*::*TIR1*::*URA3*	this study
NS272	*MATa LCB2-TAP-AID-6FLAG*::*TIR1*::*URA3*	this study
NS274	*MATa AUR1-TAP-AID-6FLAG*::*TIR1*::*URA3*	this study
NS276	*MATa KEI1-TAP-AID-6FLAG*::*TIR1*::*URA3*	this study
NS284	*MATa VRG4-TAP-AID-6FLAG*::*TIR1*::*URA3*	this study
NS269	*MATa LCB1-TAP*::*HIS3*	[15]
NS271	*MATa LCB2-TAP*::*HIS3*	[15]
NS273	*MATa Aur1-TAP*::*HIS3*	[15]
NS275	*MATa KEI1-TAP*::*HIS3*	[15]
NS283	*MATa VRG4-TAP*::*HIS3*	[15]
NS250	*MATa VRG4(A286T)*::*URA3 (from K1251)*	this study
BG2	*C. glabrata*	[41]
CAI4	*C. albicans*	[42]

The pAIDA2(6FLAG) plasmid was derived from plasmid pNHK53 (Morawska and Ulrich 2013) which bears the URA3 selectable marker and OsTIR1 expression cassette as follows. pNHK53 was digested with *Xma*I plus AscI to remove a 8 bp segment of the polylinker and then ligated to a similarly digested 1,519 bp fragment of synthetic DNA that contained the transcription terminator from the *HIS3* homologous gene of *Schizosaccharomyces pombe*, a unique MfeI cleavage site, a TAP-AID*-6FLAG coding segment identical to a portion of pHYG-AID*-6FLAG (Morawska and Ulrich 2013), a stop codon and unique *Afl*II cleavage site, and a transcription terminator from the *CYC1* gene of *S. cerevisiae* (see Figure 1A). The AID* segment within pAIDA2(6FLAG) was later removed and replaced with a codon-optimized AID* segment by digestion with BsiWI plus *Afl*II and ligation of a synthetic DNA fragment. Both plasmids and their sequences are available upon request.

To identify mutants of *S. cerevisiae* that confer resistance to SDZ 90-215, the haploid and diploid BY4741∆8 strains (Hoepfner *et al.* 2012) lacking eight genes involved in drug resistance (*SNQ2*, *PDR5*, *YOR1*, *PDR1*, *PDR2*, *PDR3*, *YAP1*, *YRM1*) were mutagenized with 2.5% ethylmethanesulfonate to 20% viability and then 2x10^7^ viable cells were plated onto SCD agar medium containing 2.5 µM SDZ 90-215. After 2 days incubation at 30°, 40 and 20 independent colonies were picked, re-tested, and subjected to whole genome sequencing as described previously (Hoepfner *et al.* 2012). Single-end reads were mapped to the S288C reference genome using BWA. All sixty SDZ 90-215-resistant strains contained either a C29Y (TGT to TAT) or a A286T (GCT to ACT) substitution mutations in the gene encoding Vrg4. No other mutations were found in the *VRG4* coding sequences, and the overall mutation rate was estimated at less than 1/50,000.

### Determination of IC50 and synergy

All chemical compounds were obtained from Sigma-Aldrich Inc. apart from aureobasidin A (Takara Inc.) and SDZ 90-215 (gift of Novartis Inc.). The IC50 of *S. cerevisiae* strains was determined by diluting stationary phase cultures 1000-fold into fresh SCD medium containing twofold serial dilutions of the indicated compounds in 96-well dishes (200 µL final volume), incubating at 30° for 24 hr, then resuspending and measuring optical density at 650 nM using a Thermomax microplate reader (Molecular Devices). Raw data were fit to the standard sigmoid equation y = ODmin + (ODmax – ODmin)/(1+(x/IC50)^slope) by non-linear regression with Kaleidagraph software. The four parameters were tabulated and the IC50s from three replicate experiments were averaged. In checkerboard assays where strains were exposed to two serially diluted compounds simultaneously, the IC50’s for each compound alone were determined as described above and then the combination index (CI) was calculated as the IC50 from the diagonal of optical densities that contain approximately 1:1 ratio of the two compounds in terms of IC50 units (Lehár *et al.* 2009). When CI equals 1, the two compounds behave additively as if they were the same compound acting on the same target. When CI is significantly less than 1, the two compounds exhibit synergy. Compounds with the same target or different targets within a single pathway often exhibit synergy (Lehár *et al.* 2007).

### Western blotting and in-gel invertase assays

For western blotting, cells were lysed via fast alkaline lysis in 0.5 M NaOH with 1.85% β-mercaptoethanol (BME) in ice water for 10 min. The protein was then precipitated by addition of trichloroacetic acid (TCA) to a final concentration of 10% (w/w) and washed once with water. Protein pellets were suspended in SDS sample buffer (0.1 M Tris-HCl, pH 6.8, 4% SDS, 0.2% bromophenol blue, 20% glycerol, 2% BME), incubated at 37° for 30 min, separated on 10% polyacrylamide gels with SDS, transferred to Hybond, and then blocked and probed with antibodies as described previously (Mehta *et al*. 2009). Primary antibodies were used as follows: anti-TAP rabbit polyclonal antibodies (ThermoFisher, CAB1001) at 1:10,000 dilution, anti-hemagglutinin (HA) mouse monoclonal antibodies (Covance, Princeton, NJ) at 1:10,000 dilution, or anti-phospho S6 (Ser-235/236) mouse monoclonal antibodies (Cell Signaling, Danvers, MA) at 1:5000 dilution, anti-PGK1 mouse monoclonal antibodies (Abcam) at 1:10,000.

In-gel invertase assays were adapted from (Huffaker and Robbins 1982). Briefly, log-phase cultures in YPD medium plus 500 mM KCl were pelleted, washed, and suspended in the same medium but with reduced glucose (0.05%) to induce invertase expression. SDZ 90-215 was added to varying concentrations and cultures were incubated at 30° for 3 hr. The cells were pelleted, washed once with 20mM sodium azide and once with TPB buffer (8.25 mM Tris-HCl pH 7, 30 mM diethylbarbituric acid, 0.1 mM phenylmethanesulfonyl fluoride) before being resuspended in 20 µL TPB buffer. Acid-washed glass beads were added to the cells before vortexing to break the cells before 50 μL of TPB buffer containing 15% glycerol and 0.01% bromophenol blue was added. The samples were centrifuged, and the supernatant was loaded onto pre-cast 10% polyacrylamide gel lacking SDS (Bio-Rad). After electrophoresis with Tris-borate buffer (8.25 mM Tris base, 80 mM boric acid, pH 7.5), the gel was incubated for 10 min in cold sucrose solution (0.1 M sucrose in 0.1 M sodium acetate, pH 5.1) at 4°. The gel was then transferred to sucrose solution at 37° and incubated for another 10 min. The gel was rinsed twice with water, and transferred to a Pyrex dish, where TTC solution (1 mg/mL 2,3,5-triphenyltetrazolium chloride in 0.5 M NaOH) was added and brought to boiling on a hot plate in the hood until color developed. Finally, the gel was washed with water and then 10% acetic acid.

### Docking of SDZ 90-215 to Vrg4

The structures of SDZ 90-215 (CID = 158437) and Vrg4 (PDB = 5OGE) were downloaded from NCBI databases, uploaded into SwissDock (Grosdidier *et al.* 2011), and modeled using default parameters. The lowest free energy models were visualized using UCSF Chimera v1.13 (Pettersen *et al.* 2004).

### Statistical tests of significance

Student’s T-tests were implemented on many datasets, as indicated in the figures (* = *P* < 0.05, ** = *P* < 0.01, *** = *P* < 0.001).

### Data Availability

All yeast strains, plasmids, and raw data are available from the corresponding author by request. File S1 contains the raw data used to generate Figure 1. Supplemental material available at Figshare: https://doi.org/10.25387/g3.7584434.

## Results

### Off-target and on-target effects of auxin and NAA in S. cerevisiae

To study the effectiveness of AID-tagging as a tool for functional genomics, we first converted the C-terminal TAP-tags (Ghaemmaghami *et al.* 2003) on 758 essential genes and 313 non-essential genes of *S. cerevisiae* into AID-tags using a one-step gene replacement strategy (Figure 1A). Briefly, the stop codon, transcription terminator, and *HIS3* selectable marker in each TAP-tagged strain was replaced with a short spacer, a minimal AID-tag, a 6xFLAG-tag with stop codon, a transcription terminator, an OsTIR1 expression cassette, and a *URA3* selectable marker. Each AID-tagged strain was purified, validated, arrayed in 96-well dishes, and stored frozen until use. To determine sensitivity to auxin and NAA, the arrayed collection was grown overnight at room temperature in SCD medium containing 0, 25, 100, or 400 µM auxin or 100 µM NAA (a non-metabolizable auxin analog) and the optical density was quantified in a microplate reader at 650 nm after 20 hr and 40 hr incubation at room temperature (ranked in Supplementary Table 1). At 25 µM auxin, growth was significantly inhibited in approximately 66% of essential and 16% of non-essential AID-tagged proteins relative to 0 µM auxin (Figure 1B), and these percentages increased slightly as auxin was increased to 100 or 400 µM or when instead 100 µM NAA was used (Figure 1C). This finding suggests that 25 µM auxin was sufficient to deplete most essential AID-tagged proteins and higher concentrations or analogs of auxin only rarely increased susceptibility.

By focusing first on just the non-essential AID-tagged proteins, which were not expected to respond to auxin, we noticed that 25, 100, and 400 µM auxin progressively lowered the final cell density in the stationary phase cultures, with 100 µM NAA as the most toxic of all. After a 20 hr growth period, the median optical densities declined by 2, 5, 13, and 19% (Figure 1D). These declines persisted even after 40 hr growth period (data not shown). Similar declines in yield were seen in the wild-type parental strain that lacked the AID-tag and the OsTIR1 expression cassette. Because exponential growth rate was not altered in these conditions (Nishimura *et al.* 2009; Morawska and Ulrich 2013), high auxin and NAA may have significant off-target effects as cultures approach the stationary phase.

**Figure 2:**
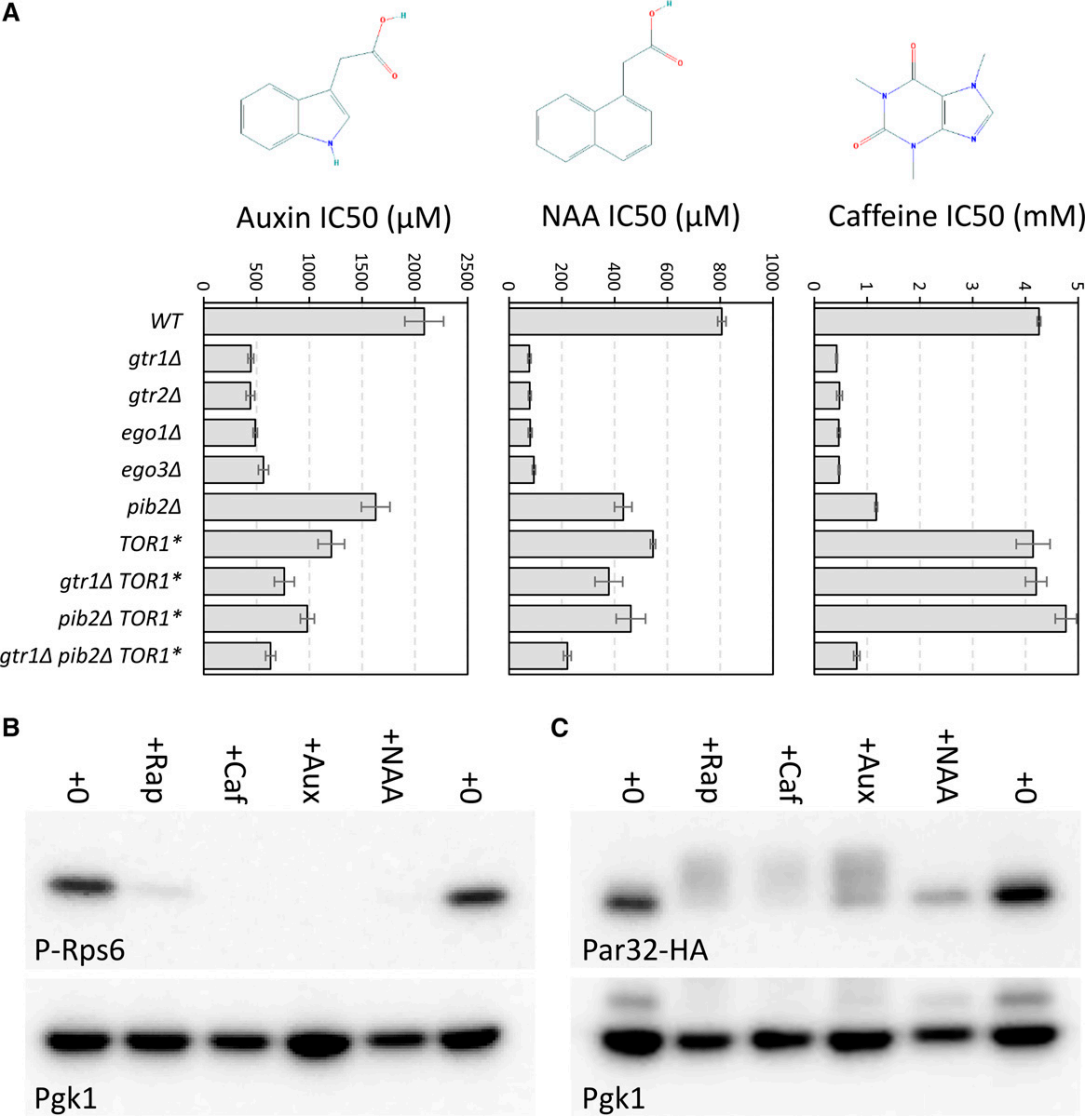
Auxin and NAA inhibit TORC1 signaling. (A) The concentrations of auxin, NAA, and caffeine that cause 50% inhibition of growth (IC50) of the indicated strains in SCD medium are shown as bars (average ± SD of 3 biological replicates). Chemical structures are shown as insets. Crude cell lysates of a wild-type (B) and wild-type expressing Par32-3xHA from plasmid pMS034 (Huber *et al.* 2009) (C) strains were prepared from log-phase cultures that had been exposed for 1 hr to 0.2 µg/mL rapamycin, 8 mM caffeine, 4 mM auxin, or 1.6 mM NAA and analyzed by western blotting using polyclonal antibodies that recognize phospho-eEF1B-α (B) or monoclonal antibodies that recognize HA (C). Blots were stripped and re-exposed to antibodies recognizing Pgk1 as a measure of gel loading.

To explore the possible off-target effects of auxin and NAA, we examined the median-normalized dataset for clear instances of selective toxicity to only the highest concentrations of auxin or to NAA. Four non-essential AID-tagged proteins (Gtr1, Gtr2, Ego1, Ego3) exhibited unusual hypersensitivity to 100 µM NAA and 400 µM auxin relative to 25 µM auxin, a pattern that was not observed elsewhere in the collection of AID-tagged strains. All four proteins bind to each other and function together as positive regulators of the rapamycin-sensitive protein kinase known as TORC1 (Loewith and Hall 2011). Conversely, a negative regulator of this GTR-EGO complex (Npr2) exhibited significant resistance to high auxin and NAA when AID-tagged. One possible explanation for these findings is that TORC1 itself can be inhibited by auxin and NAA, and depletion of the GTR-EGO proteins thus causes hypersensitivity to inhibitors of TORC1. To test this idea, the sensitivities of *gtr1∆*, *gtr2∆*, *ego1∆*, and *ego3∆* knockout mutants to auxin and NAA were evaluated in growth assays spanning a large range of concentrations (Figure 2A). All four EGO-GTR-deficient knockout mutants exhibited strong hypersensitivity to auxin (3.7- to 4.sevenfold) and NAA (8.6- to 10.fivefold) relative to the wild-type parent strain BY4741. These effects of auxin and NAA were similar to those of caffeine, a direct inhibitor of TORC1 activity (Reinke *et al.* 2006). A single amino acid substitution (Tor1-S1954L) that hyperactivates TORC1 (Kim and Cunningham 2015) largely reversed the hypersensitivity of *gtr1∆* mutants to auxin, NAA, and caffeine in *gtr1∆* strains to almost the same level as the strain expressing Gtr1 (Figure 2A). Auxin and NAA probably do not inhibit Pib2, a positive regulator of TORC1 that operates independent of GTR-EGO (Kim and Cunningham 2015; Michel *et al.* 2017; Tanigawa and Maeda 2017; Varlakhanova *et al.* 2017), because the *pib2∆* knockout mutants still exhibited mild hypersensitivity to auxin and NAA as well as caffeine (Figure 2A) as expected for mildly reduced TORC1 activity. To test whether auxin and NAA can inhibit TORC1 function, the phosphorylation of two indirect targets of TORC1 (Rps6 (González *et al.* 2015) and Par32 (Huber *et al.* 2009)) was measured by western blotting after 1 hr incubation of wild-type cells with these compounds. Auxin and NAA strongly decreased phosphorylation of Rps6 similar to caffeine and rapamycin (Figure 2B). Auxin increased phosphorylation of Par32-HA similar to caffeine and rapamycin but in this case NAA seemed less inhibitory than auxin (Figure 2C). Collectively, these findings suggest that auxin and NAA can inhibit TORC1 itself or TORC1-dependent proliferation.

Five non-essential proteins involved in biosynthesis of serine and threonine (Hom2, Hom3, Hom6, Ser2, Thr4) also exhibited slow growth rate when AID-tagged and exposed to auxin. Because *hom2∆*, *hom3∆*, *hom6∆*, *ser2∆*, and *thr4∆* mutations exhibit strongly fitness defects when combined with *gtr1∆*, *ego1∆*, or *pib2∆* mutations (Costanzo *et al.* 2010), it is possible that off-target effects of auxin on TORC1 contribute to growth defects of the AID-tagged strains. But off-target effects on TORC1 are not likely to explain why 39 additional non-essential AID-tagged proteins exhibited growth inhibition at 25 µM auxin because these proteins did not exhibit genetic interactions with TORC1 regulators and did not exhibit the characteristic of increased growth inhibition at 100 µM NAA or 400 µM auxin. Interestingly, many of these responders can be grouped by function and structure. For example, two proteins required for biosynthesis of cysteine (Cys3, Cys4) exhibited slow growth rate when AID-tagged and exposed to auxin even though cysteine was present in SCD culture medium. Additionally, eight of eleven AID-tagged proteins that form the non-essential V-ATPase exhibited slow growth in low auxin. Last, two non-essential subunits (Cog1, Cog8) and one essential subunit (Cog4) of the octomeric COG complex exhibited sensitivity to 25 µM auxin when AID-tagged, whereas the other subunits did not. These observations may help expose additional off-target effects of auxin and NAA that are potentially independent of TORC1. Alternatively, they may reveal transient inhibition of growth upon depletion of the target that endures until adaptive processes become engaged and compensate for a sudden metabolic disruption.

When the AID-tag is sequestered within membrane-bound organelles, the tagged protein is expected to be inaccessible to the ubiquitin proteasome system and thus insensitive to auxin. Of 32 essential proteins with C-termini localized within mitochondria, only 2 AID-tagged strains (Tim50, Tim17) were significantly sensitive to low auxin. Of 16 essential proteins with C-termini localized within the lumens of the endoplasmic reticulum or Golgi complex, 5 strains (Brr6, Stt3, Cdc1, Tre2, Smx3) were sensitive to auxin when AID-tagged. The few exceptions may become susceptible to depletion at some point during biogenesis before the C-terminus is sequestered or may have incorrectly assigned topologies. When these 48 proteins are excluded, the overall susceptibility of essential proteins to depletion with auxin rose from 66 to 70%.

Interestingly, 42 out of 53 essential transmembrane proteins of the secretory pathway with C-termini facing the cytoplasm (79%) exhibited significant sensitivity to auxin after AID-tagging. This group includes polytopic transmembrane proteins that localize to the plasma membrane (Alr1, Pma1, Hip1), the Golgi complex (Vrg4, Kei1, Lsm8), the nuclear pore complex that spans the nuclear envelope (Nup192), as well as numerous essential proteins of the endoplasmic reticulum which contains numerous essential proteins that participate in biosynthesis of lipids and glycoproteins. Additionally, 3 essential proteins associated with the mitochondrial outer membrane and with C-termini exposed to the cytoplasm (Sen2, Sen54, Sam35) also conferred sensitivity to auxin when AID-tagged, while 2 others (Tom20, Tom22) did not. Excluding the secretome and mitochondriome, we find 450 out of 652 (69%) of the remaining AID-tagged essential proteins exhibited significant sensitivity to auxin, a population that included proteins in the cytoplasm, nucleus, nucleolus, spindle pole body, as well as peripheral associations with the membrane-bound organelles listed earlier (Supplemental Table 1). Using GOrilla (Eden *et al.* 2009), we tested whether the set of 202 essential proteins that did not produce growth phenotypes when AID-tagged was significantly enriched with any biological process, function or component. Only two processes were returned. The most significant process was labeled “proteasomal ubiquitin-independent protein catabolic process” (GO:0010499; P-value = 6.6E-6, FRD q-value = 1.6E-2, enrichment = 3.25) that was represented by ten core subunits of the 20S proteasome (Pre1, Pre2, Pre3, Pre4, Pre5, Pre6, Pre8, Pre10, Pup2, Scl1). Because the 20S proteasome is necessary for depleting its own core subunits, their depletion is potentially self-limiting before reaching a phenotypic threshold. The second process represented by 183 genes was labeled “organonitrogen compound metabolic process” (GO:1901564; P-value = 6.9E-4, FDR q-value = 8.4E-1, enrichment = 1.31). These data show that auxin can frequently induce depletion of essential AID-tagged proteins to levels that become growth limiting as long as the tag is exposed to the cytoplasm or nucleoplasm. A minority of AID-tagged essential proteins did not exhibit growth inhibition by auxin in these conditions for unknown reasons.

### TAP-tags and AID-tags can dampen protein expression and function

**Figure 3:**
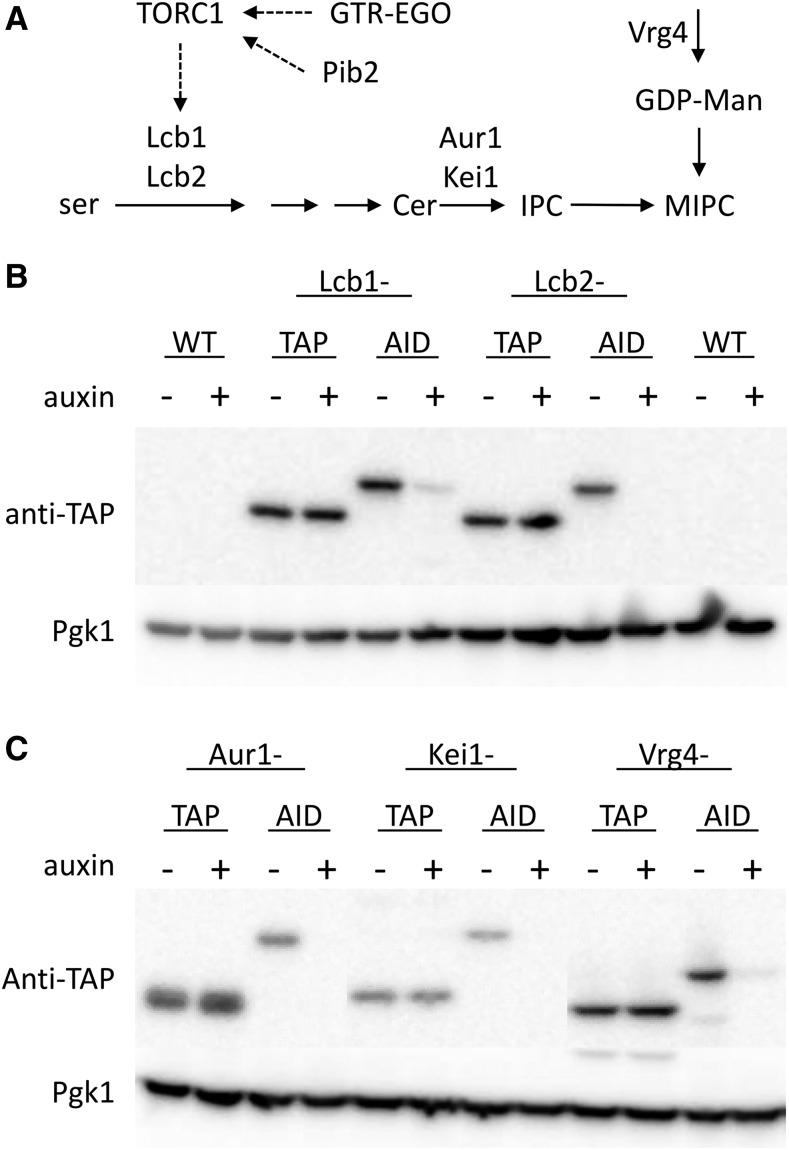
Transmembrane proteins involved in sphingolipid biosynthesis are susceptible to depletion. (A) Schematic representation of sphingolipid biosynthesis pathway from serine (ser) to ceramide (Cer) to inositolphosphoryl-ceramide (IPC) to mannosyl-IPC (MIPC) from luminal GDP-mannose is depicted along with proteins that catalyze the reactions and their regulators (TORC1, Pib2, GTR-EGO). (B,C) A polyclonal antibody that recognizes a common segment of TAP- and AID-tags was used in western blot analyses of the indicated strains that had been grown to log phase and exposed to 100 µM auxin for 1 hr as indicated.

The effects of auxin on expression of several AID-tagged and TAP-tagged essential transmembrane proteins involved in sphingolipid biosynthesis (Figure 3A) were investigated by western blotting (Figure 3B-3C). Surprisingly, in the absence of auxin, AID-tagging slightly, but reproducibly, diminished log-phase expression of Lcb1, Lcb2, Aur1, Kei1, and Vrg4 relative to the TAP-tagged parent strains. The lower expression of AID-tagged proteins relative to TAP-tagged controls may explain why ∼8% of the TAP-tagged essential proteins were not successfully AID-tagged. The addition of 100 µM auxin for 60 min strongly depleted the AID-tagged variants but not the TAP-tagged proteins. These findings suggest that AID-tags confer sensitivity to auxin even for transmembrane proteins in the endoplasmic reticulum (Lcb1, Lcb2) and Golgi complex (Aur1, Kei1, Vrg4) and can slightly dampen expression of the attached protein relative to the parental TAP-tags.

**Figure 4:**
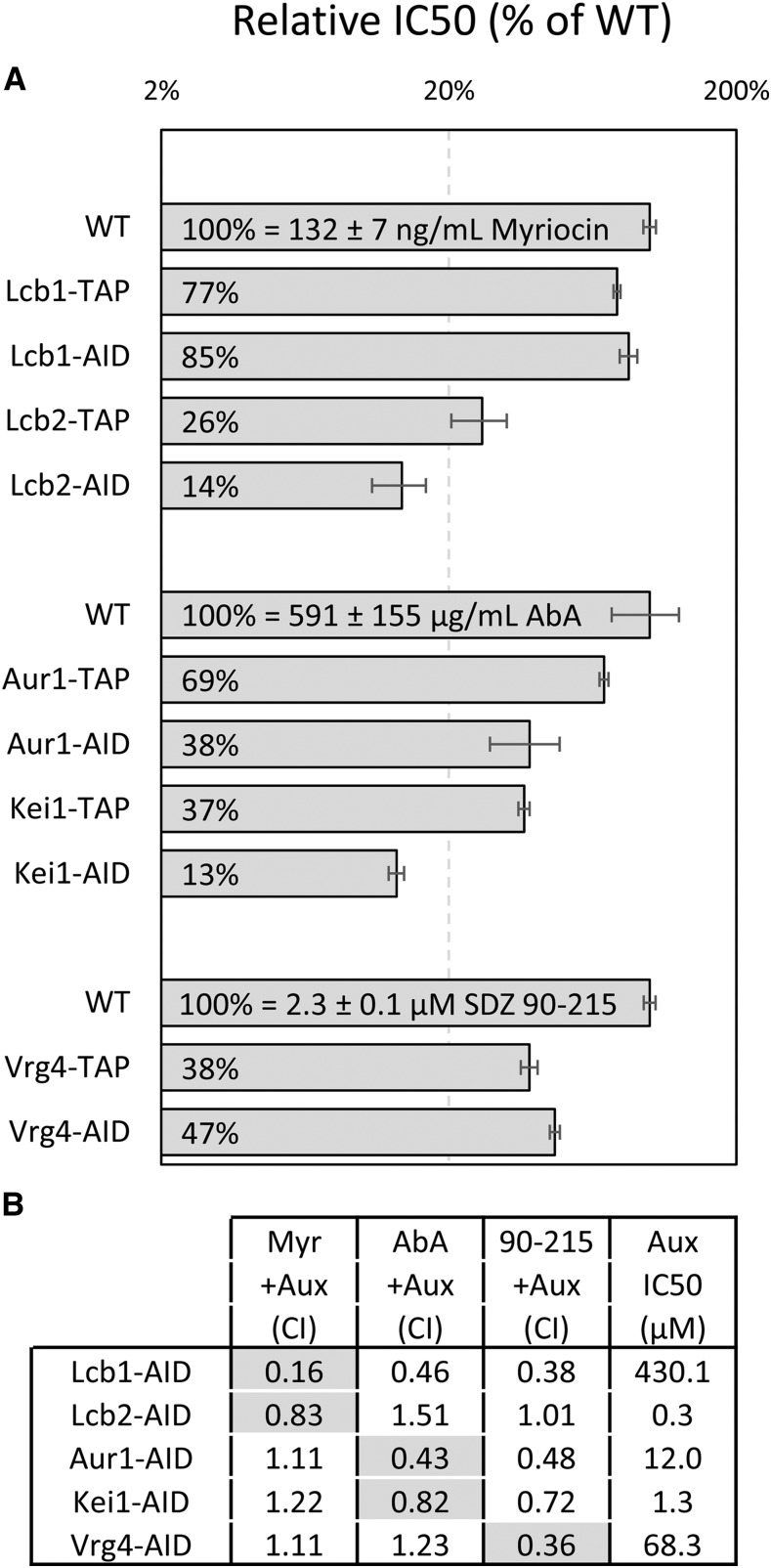
TAP- and AID-tagged become hypersensitive to on-target inhibitors that can synergize. (A) Three replicate cultures of the indicated strains were diluted into fresh SCD medium containing varying concentrations of myriocin, aureobasidin A (AbA) or SDZ 90-215 and average IC50 was charted relative to the wild-type control strain (± SD). (B) The IC50 for auxin was determined for several AID-tagged strains, and the combination index (CI) with myriocin, aureobasidin A, and SDZ 90-215 was determined from checkerboard assays. Synergy is evident when CI is less than 1. Shaded boxes indicate on-target inhibition when synergy was expected.

To investigate the degree of functional dampening by AID-tags, we quantitatively measured the hypersensitivities of AID-tagged and TAP-tagged strains to known on-target inhibitors. Myriocin blocks the essential enzyme serine palmitoyltransferase that is composed of essential proteins Lcb1 and Lcb2 (Nagiec *et al.* 1994) and *lcb1∆*/+ and *lcb2∆/+* heterozygous diploid strains are more sensitive to myriocin than +/+ wild-type diploid strains in growth assays (Hillenmeyer *et al.* 2008; Hoepfner *et al.* 2014; Lee *et al.* 2014). Interestingly, the concentration of myriocin that causes a 50% decrease in growth (the IC50) was diminished slightly (by 1.3- and 1.twofold) in Lcb1-TAP and Lcb1-AID strains and diminished strongly (by 3.8- and 7.3- fold) in Lcb2-TAP and Lcb2-AID strains, respectively, relative to the wild-type parent strain (Figure 4A). Similarly, resistance to aureobasidin A was diminished moderately (by 1.4- and 2.sixfold) in Aur1-TAP and Aur1-AID strains and diminished very strongly (by 17- and 29-fold) in Kei1-TAP and Kei1-AID strains, respectively (Figure 4A). Aureobasidin A inhibits the enzyme inositolphosphoryl-ceramide synthase of the Golgi complex that is composed of essential Aur1 and Kei1 proteins (Hashida-Okado *et al.* 1996; Sato *et al.* 2009). Last, resistance of Vrg4-TAP and Vrg4-AID strains to SDZ 90-215, a novel compound that targets the essential GDP-mannose transporter of the Golgi complex (see next section), was moderately diminished (by 2.6- and 2.onefold). In summary, TAP-tagging alone caused hypersensitivity to on-target inhibitors by 1.3- to 17-fold while AID-tagging conferred additional hypersensitivity of ∼1.eightfold in three of five instances. Though C-terminal TAP-tagging was shown to have little effect on expression of client proteins (Ghaemmaghami *et al.* 2003), our findings suggest the TAP-tag often diminishes their function. Thus, TAP-tagged and AID-tagged proteins often behave like untagged “DAmP” strains that lack a 3′ transcription terminator (Breslow *et al.* 2008), which usually decreases mRNA stability and thus protein expression.

The AID-tagged essential proteins generated here may allow tunable dampening of expression at sub-phenotypic concentrations of auxin, and thus may offer new opportunities for discovery and characterization of novel on-target small molecule inhibitors. We tested this possibility using AID-tagged variants of Lcb1, Lcb2, Aur1, Kei1, and Vrg4 that were simultaneously exposed to varying concentrations of auxin and varying concentrations of myriocin, aureobasidin A, and SDZ 90-215 that span the IC50 for each strain. From these checkerboard assays, the IC50 of each drug alone can be measured for each strain and also the degree of synergism can be estimated by calculating the combination index (CI; see Methods). A CI less than 1 indicates synergism, a CI equal to 1 indicates additivity, and a CI greater than 1 indicates antagonism between the two tested compounds. For Myriocin and auxin, strong synergism was observed for the Lcb1-AID strain (CI = 0.16), weak synergism was observed for the Lcb2-AID strain (CI = 0.83), and no synergism was observed for the Aur1-, Kei1-, and Vrg4-AID strains (CI > 1; Figure 4B). Aureobasidin A and auxin synergized on the Aur1- and Kei1-AID strains (CI = 0.43 and 0.82) but not the Lcb2- and Vrg4-AID strains (CI = 1.51 and 1.23). SDZ 90-215 synergized with auxin on the Vrg4-AID strain (CI = 0.36) and not the Lcb2-AID strain (CI = 1.01). Though aureobasidin A and SDZ 90-215 both synergized with auxin for several off-target AID-tagged proteins, all three molecules synergized with auxin for all their on-target AID-tagged strains. Thus, low concentrations of auxin can be used to sensitize many AID-tagged strains to on-target inhibitors.

### SDZ 90-215 may target Vrg4

**Figure 5:**
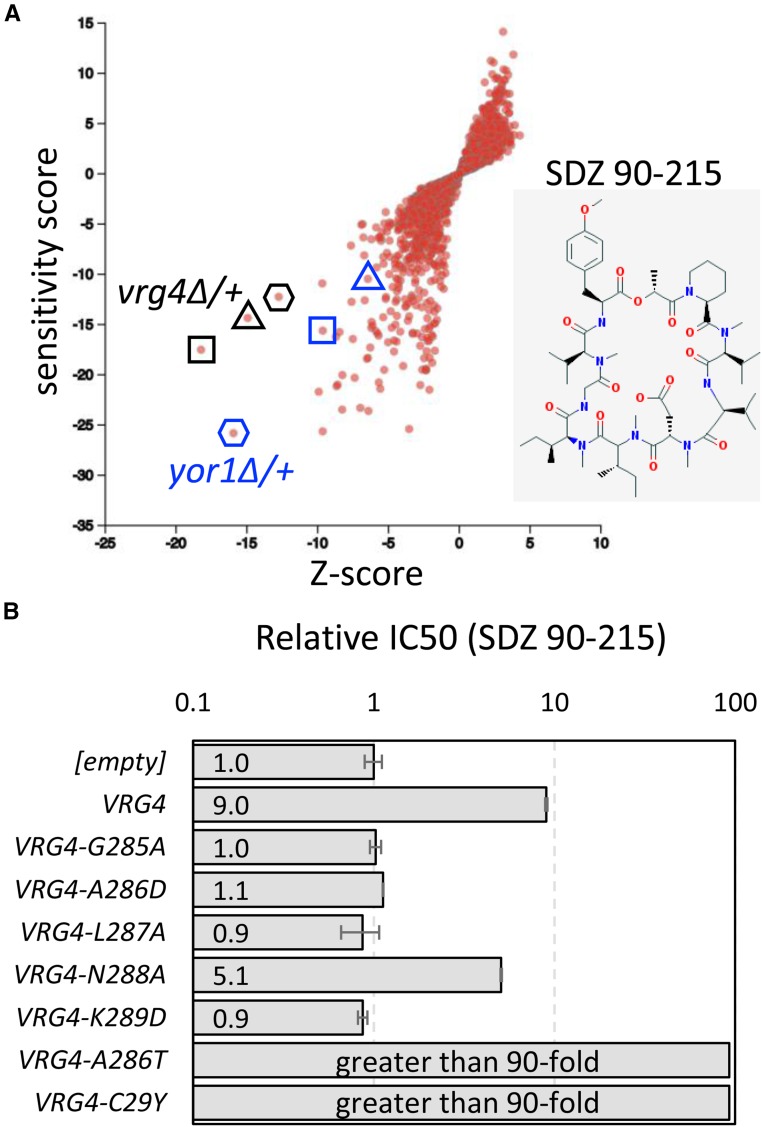
SDZ 90-215 may target Vrg4. (A) The responses of each heterozygous knockout mutant of *S. cerevisiae* to 3 (triangle), 4 (square), and 6 (hexagon) µg/mL SDZ 90-215 (inset) was charted as a function of Z-score and sensitivity score as described previously (Hoepfner *et al.* 2014). The heterozygous *vrg4∆/+* and *yor1∆/+* mutant strains are circled. (B) A wild-type strain was transformed with high-dosage plasmids bearing *VRG4* or several derivatives and the relative IC50 to SDZ 90-215 was determined (empty plasmid set to 1.0). Bars represent the average ± SD from three biological replicates.

SDZ 90-215 is a cyclic peptide secreted by *Septoria* species that exhibits strong antifungal activity toward several pathogenic yeasts (Emmer *et al.* 1994), but the essential target of SDZ 90-215 has not been identified. In a large chemical genomics screen involving thousands of compounds and a panel of heterozygous knockout mutants (Hoepfner *et al.* 2014), only the *vrg4∆/+* and *yor1∆/+* diploid strains of *S. cerevisiae* were significantly hypersensitive to SDZ 90-215 (Figure 5A). Yor1 is a non-essential multidrug transporter in the plasma membrane that mediates efflux of many organic anions from the cytoplasm. The *yor1∆/∆* diploids were also extremely hypersensitive to SDZ 90-215 (Hoepfner *et al.* 2014), suggesting that Yor1 promotes export of the compound from the cytoplasm. Vrg4 is an essential GDP-mannose transporter in the Golgi complex (Dean *et al.* 1997) and a good candidate for inhibition by SDZ 90-215. As mentioned earlier, the haploid Vrg4-TAP and Vrg4-AID strains were hypersensitive to SDZ 90-215 in growth assays (Figure 4A). Also, the Vrg4-AID strain was synergistically affected by auxin and SDZ 90-215 but not Myriocin or Aureobasidin (Figure 4B). Last, overexpression of Vrg4 from a high dosage plasmid (YEpGAP-VRG4) increased resistance to SDZ 90-215 by ninefold relative to an empty control plasmid (YEpGAP) whereas overexpression of catalytically inactive variants of Vrg4 (G285A, A286D, L287A, K289D substitutions) (Gao *et al.* 2001) did not alter resistance and a partially inactive variant (N288A) caused weak resistance (Figure 5B).

**Figure 6:**
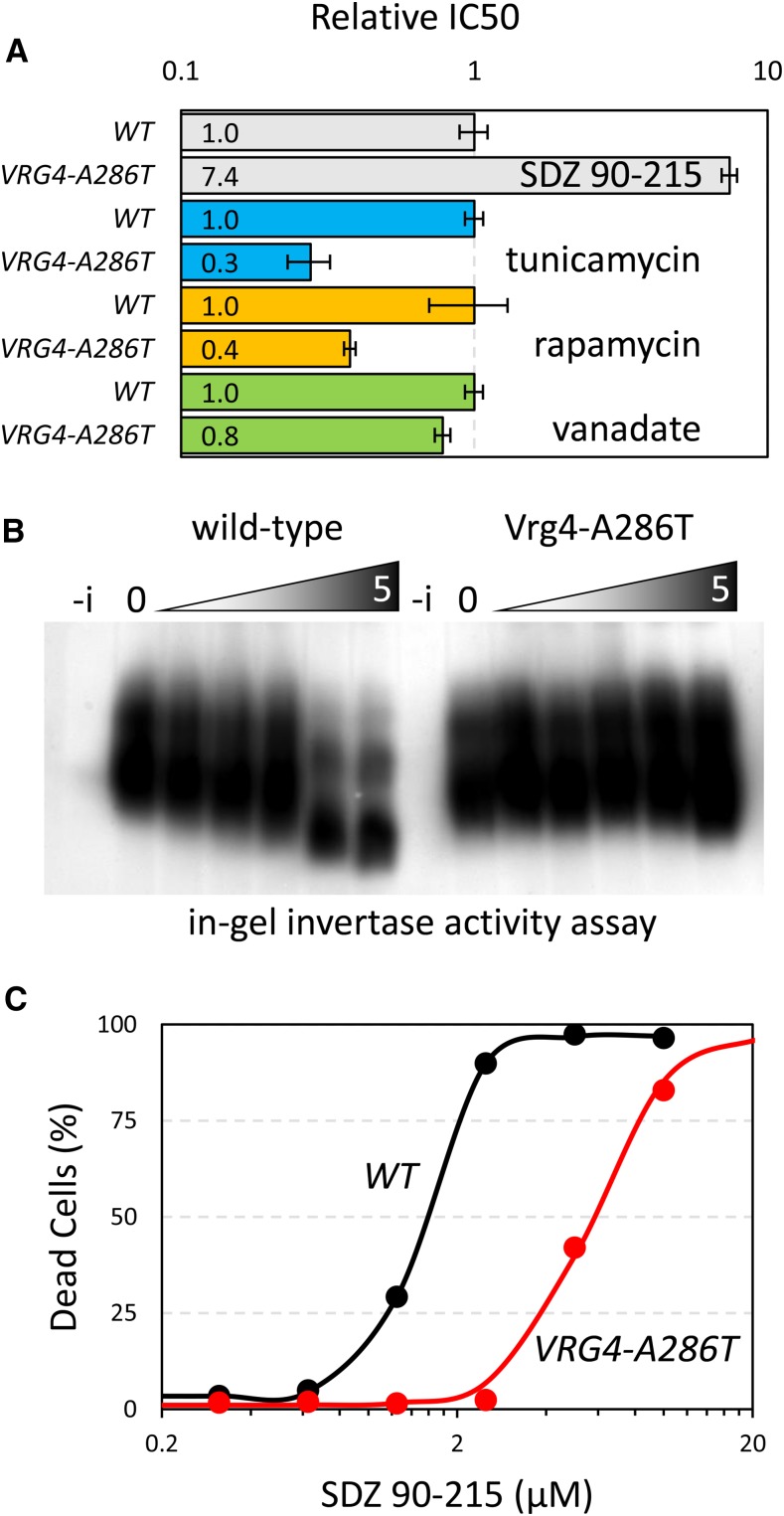
The function of Vrg4-A286T specifically resists SDZ 90-215. (A) The Vrg4-A286T mutation was introduced into the wild-type B4741 strain background and the IC50’s of SDZ 90-215 (gray), tunicamycin (blue), rapamycin (orange), and sodium ortho-vanadate (green) were determined in both strains. Bars represent the averages ± SD from three biological replicates with wild-type set to 1.0. (B) The SDZ 90-215-resistant Vrg4-A286T strain and wild-type control strain were grown to log phase in SCD medium, then shifted to the same medium (-i) or to invertase-inducing medium containing sucrose instead of glucose that also contained 0 to 5 µM SDZ 90-215. After 3 hr, lysates were prepared, subjected non-denaturing PAGE, and stained for invertase activity. Faster migrating bands represent hypo-glycosylated forms. (C) The wild-type and Vrg4-A286T strains were diluted into fresh SCD medium containing the indicated concentrations of SDZ 90-215, incubated for 24 hr at 30°C, then stained with propidium iodide and analyzed by flow cytometry for live and dead cells. Dots represent average of 3 biological replicates and best-fit curves represent non-linear regression to the standard sigmoid equation. The 50% lethal dose differed by almost fourfold.

If SDZ 90-215 directly inhibits Vrg4, it may be possible to isolate Vrg4 variants that are catalytically active but resistant to SDZ 90-215. To test this possibility, a haploid and a diploid yeast strain lacking Yor1 and seven other drug-resistance proteins were mutagenized and plated on medium containing 2.5 µM SDZ 90-215. Of 60 independent colonies that proved resistant to SDZ 90-215, 24 expressed a C29Y variant of Vrg4 and 36 expressed an A286T variant of Vrg4 (all G/A transitions). When integrated into the high-copy plasmid-based *VRG4* gene, the overexpressed C29A and A286T variants increased resistance to SDZ 90-215 by over 10-fold relative to the wild-type Vrg4 plasmid (Figure 5B). Integration of the A286T substitution mutation into the *VRG4* locus of wild-type cells increased resistance to SDZ 90-215 by 7.fourfold as expected, but did not increase resistance to tunicamycin, rapamycin, or sodium ortho-vanadate (Figure 6A). These findings suggest that the mutation does not cause general drug resistance. SDZ 90-215 also caused a dose-dependent inhibition of outer chain mannosylation of a secreted protein (invertase) in wild-type cells, but not in Vrg4-A286T mutant cells, as indicated by alterations in native gel mobility (Figure 6B). The Vrg4-A286T was also nearly fourfold more resistant to SDZ 90-215 in measurements of cell death (Figure 6C). Collectively, these findings suggest that SDZ 90-215 may directly engage Vrg4 from the cytoplasm and may inhibit its essential functions.

**Figure 7:**
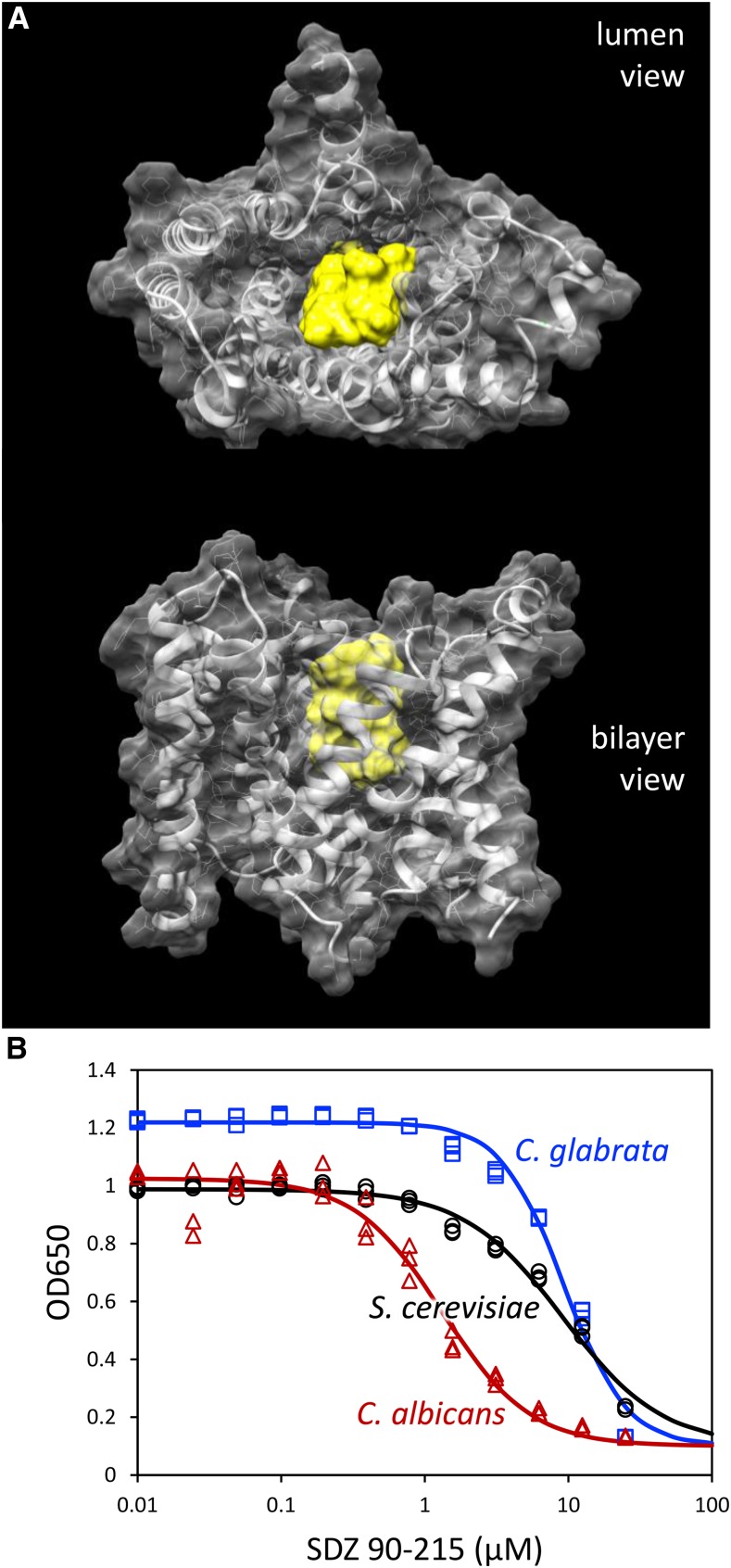
SDZ 90-215 can dock onto Vrg4 from *S. cerevisiae* and inhibit growth of pathogenic *Candida* species. (A) A model of SDZ 90-215 (yellow) docked into the large central cavity of Vrg4 (translucent gray) was generated using SwissDock and viewed from the Golgi lumen or the plane of the bilayer. (B) Wild-type strains of *S. cerevisiae* BY4741, *C. glabrata* BG2 (Cormack and Falkow 1999), and *C. albicans* CAI4 (Fonzi and Irwin 1993) were diluted into fresh YPD containing the indicated concentrations of SDZ 90-215 then optical density at 650 nm was measured after 24 hr. The symbols represent 3 biological replicates and the best-fit curves were obtained by non-linear regression to the standard sigmoid equation.

Vrg4 is a cup-shaped transmembrane protein with a large central substrate-binding cavity that opens alternately to the Golgi lumen and to the cytoplasm (Parker and Newstead 2017). In both conformations, C29 and A286 were positioned near the base of the cavity in the middle of transmembrane helices 1 and 9. C29 is adjacent to a conserved and functionally critical Y28 residue that makes direct contact with the ribose portion of the substrate GDP-mannose deep within the cavity (Parker and Newstead 2017). A286 is one turn of helix 9 away from K289, which contacts the second phosphate of GDP-mannose and is part of the signature GALNK motif conserved among mannose-selective nucleotide sugar transporters. The A286T and C29Y substitutions may therefore cause resistance to SDZ 90-215 by diminishing affinity of the inhibitor without abolishing affinity of substrates. To determine whether SDZ 90-215 (1,126 D) can plausibly fit within the substrate-binding cavity, the structures of SDZ 90-215 and Vrg4 (open to the lumen) were uploaded into SwissDock and modeled. SwissDock generated models where SDZ 90-215 occupies nearly all of the central cavity of Vrg4 (Figure 7A) with a favorable free energy (∆G = -9.04). SwissDock generated similarly favorable models of GDP-mannose deep within the central cavity of Vrg4 (∆G = -9.35) that closely resembled the experimentally determined structure. Though SDZ 90-215 likely engages Vrg4 from the cytoplasm instead of the Golgi lumen, the central cavity is thought to be of similar size and shape when open to the cytoplasm owing to an internal duplication and topological inversion of a domain within the protein (Parker and Newstead 2017). Therefore, SDZ 90-215 may compete with GDP-mannose (605 D) for binding in the central cavity of Vrg4, causing decreased mannosylation of sphingolipids and glycoproteins, slowed cell proliferation, and eventual cell death. However, direct measurementsof binding and transport measurements will be required to confirm this hypothesis.

SDZ 90-215 was found previously to inhibit *in vitro* growth of the pathogenic yeasts *Candida albicans*, *C. tropicalis*, *C. guilliermondii*, and *C. krusei* and also to cure several types of *C. albicans* infections in mouse models without toxicity to the animals (Emmer *et al.* 1994). To compare and extend those findings, we determined the IC50’s of SDZ 90-215 on *S. cerevisiae* (strain BY4741) and *C. glabrata* (strain BG2) in addition to *C. albicans* (strain CAI4). In YPD culture medium at 30°, both *S. cerevisiae* and *C. glabrata* exhibited approximately sevenfold increase in IC50 relative to that of *C. albicans* (IC50 = 1.33 µM; Figure 7B). Thus, SDZ 90-215 may be useful as a lead compound for treatment of yeast infections. Collectively, these findings demonstrate the utility of AID-tagging and auxin-induced depletion of target proteins as both a tool for discovery of new SMIs and a means of modeling the actions of SMIs before they have been discovered.

## Discussion

This study provides strong evidence that auxin-induced depletion of AID-tagged target proteins is a broadly effective strategy for analyzing protein function on a proteome-wide scale. At least 70% of essential proteins with C-termini exposed to the cytoplasm or nucleoplasm could be depleted functionally, as detected by slower growth of cultures, when tagged with AID and exposed to low concentrations of auxin (25 µM). The susceptible proteins included non-transmembrane proteins as well as polytopic transmembrane proteins in the plasma membrane, Golgi complex, endoplasmic reticulum, and mitochondrion. When C-terminal AID-tags are sequestered within these organelles, functional depletion was almost always abolished as expected due to OsTir1 localization in the cytoplasm and nucleoplasm. Among five susceptible AID-tagged essential transmembrane proteins that we studied carefully, the IC50 for auxin ranged from 0.3 to 430 µM, spanning more than 1,000-fold. The source of the variation likely involves unexpected complexity in the microenvironments surrounding AID-tags, which could vary substantially among targets. Different genes may require very different levels of depletion to produce a growth phenotype. For instance, even before adding auxin, the sensitivity of Lcb1-AID and Lcb2-AID strains to an on-target inhibitor (myriocin) was increased 1.twofold and 7.threefold, respectively, relative to untagged wild-type strain. Thus, the latter strain was likely to be closer to the phenotypic threshold. Very low auxin concentrations might deplete Lcb2-AID below the point where it limits growth while being insufficient to deplete Lcb1-AID to the phenotypic level because of its near wild-type level of function. Similar reasoning could explain why the IC50s of Kei1-AID strains for auxin and an on-target inhibitor (aureobasidin A) are ninefold and sixfold lower than those of Aur1-AID strains. The amount of auxin required to observe a growth defect in any given AID-tagged strain will depend in part on the degree of depletion required to become limiting for growth, which is target specific.

Interestingly, we observed that TAP-tagging alone diminished resistance to on-target inhibitors by 1.threefold to 2.sevenfold for the five strains studied in detail. TAP-tagging had little or no reported effect on expression (Ghaemmaghami *et al.* 2003), but clearly can have significant effects on protein function. About 25% of essential proteins could not survive with just a C-terminal TAP-tag (Ghaemmaghami *et al.* 2003) and were not studied here. AID-tagging often reduced expression of proteins by up to twofold, and a codon-optimized AID-tag usually improved basal expression (K. W. C., unpublished results). These findings suggest that many TAP- and AID-tagged essential proteins have significantly damped expression and/or function, providing a useful alternative to the collection of untagged proteins with damped expression (Breslow *et al.* 2008).

Here we report that auxin and especially NAA both have off-target effects as inhibitors of TORC1 signaling, especially at high concentrations. Knockout mutants lacking positive regulators of TORC1 became hypersensitive to known on-target inhibitors (rapamycin and caffeine) as well as auxin and NAA, while hyperactivating mutations in Tor1 largely reversed these hypersensitivities. Additionally, just 1 hr exposure to auxin or NAA altered phosphorylation of two downstream effectors of TORC1. Because NAA had greater activity against TORC1 and similar activity against AID-tagged proteins as compared to auxin, extra caution should be used when interpreting results with high concentrations of NAA. NAA exhibits much less phototoxicity in yeast cells imaged with blue light (Papagiannakis *et al.* 2017). While novel inhibitors of TORC1 and its mammalian ortholog mTORC1 are potentially useful as immunosuppressants, the AID-tagged strains generated here may be useful for discovering auxin-like analogs that have decreased off-target activities and increased selectivity for AID-tags. In any case, users of AID technologies should always attempt to minimize and detect off-target effects by varying the concentration of auxin on both AID-tagged and TAP-tagged derivatives of target proteins.

Acute depletion of AID-tagged proteins with auxin can be instrumental in deciphering gene function before secondary responses and compensatory effects become manifest (Holland *et al.* 2012). Mild depletion of AID-tagged proteins can sensitize cells to on-target inhibitors, and thus facilitate screening and discovery of small molecule inhibitors (SMIs) akin to earlier sensitization strategies that involve haploinsufficiency or damped expression. A screen of 1,776 compounds using complete collections of homozygous and heterozygous gene knockout mutants of yeast identified the essential GDP-mannose transporter Vrg4 as a possible target of SDZ 90-215 and the ABC-family transporter Yor1 as a possible SDZ 90-215 efflux pump (Hoepfner *et al.* 2014). Here we present additional data suggesting that SDZ 90-215 targets Vrg4: (1) Vrg4-TAP and Vrg4-AID strains were hypersensitive to SDZ 90-215, (2) auxin synergized with SDZ 90-215 only on the latter strain, (3) overexpression of Vrg4 caused resistance to SDZ 90-215, (4) amino acid substitutions near the substrate-binding sites of Vrg4 caused resistance to SDZ 90-215 and not off-target molecules, (5) the biological function of Vrg4 in mannosylation of a secretory protein was inhibited by exposure of cells to SDZ 90-215, and (6) SDZ 90-215 can be accommodated within the large central cavity observed in an X-ray crystal structure of Vrg4. Though the central cavity was open to the lumen of the Golgi complex in this conformation of Vrg4, the conformation that opens to the cytoplasm where SDZ 90-215 likely binds is predicted to be similar due to the internal duplication and topological inversion of a five-transmembrane domain to generate the full-length ten-transmembrane polytopic protein (Parker and Newstead 2017).

Vrg4 is essential in *S. cerevisiae*, *C. glabrata*, and *C. ablicans* for mannosylation reactions in the Golgi complex, and hence for normal synthesis of glycoproteins, mannan in the cell wall, and sphingolipids in the cell membranes (Nishikawa *et al.* 2002). Because these functions are unique to fungi, Vrg4 inhibitors are expected to exhibit antifungal properties. SDZ 90-215 (SDZ 90-215) was found previously to have potent antifungal activity against several pathogenic yeasts with low toxicity in mouse (Emmer *et al.* 1994). As a natural product synthesized by a species of filamentous fungus in the genus *Septoria*, SDZ 90-215 and related cyclopeptides may be natural antifungals that are optimized to target Vrg4 or other members of the SLC35 family of nucleotide sugar transporters.

The AID-tagged collection of essential and non-essential genes produced here adds another valuable approach for accelerating functional genomics research and for discovery of new pharmaceuticals and nutraceuticals. The system can be used to discover auxin-like with superior properties, such as higher on-target potency and lower off-target effects. Though the strains lack explicit barcodes that can facilitate experiments on pooled strains, each AID-tag is inserted in a different genomic locus that itself can be used as a unique barcode. Methods now exist for simultaneously profiling thousands of different barcode-free insertions sites in pools of cells and quantifying changes in their frequency over time in response to different drugs or culture conditions (Guo *et al.* 2013; Bronner *et al.* 2016; Michel *et al.* 2017; Segal *et al.* 2018). Auxin-induced protein depletion may also be combined in the same tag with drug-induced mRNA degradation or depletion strategies to further enhance the rate and extent of protein depletion, thus providing even tighter control of gene function at the genome-wide level.
